# Differences in Dysfunction of Thenar and Hypothenar Motoneurons in Amyotrophic Lateral Sclerosis

**DOI:** 10.3389/fnhum.2016.00099

**Published:** 2016-03-07

**Authors:** Jia Fang, Liying Cui, Mingsheng Liu, Yuzhou Guan, Xiaoguang Li, Dawei Li, Bo Cui, Dongchao Shen, Qingyun Ding

**Affiliations:** ^1^Department of Neurology, Peking Union Medical College Hospital, Chinese Academy of Medical Sciences and Peking Union Medical CollegeBeijing, China; ^2^Neuroscience Center, Chinese Academy of Medical SciencesBeijing, China

**Keywords:** amyotrophic lateral sclerosis, F-wave, abductor pollicis brevis, abductor digiti minimi, motor neuron, split-hand

## Abstract

This study aimed to determine differences in spinal motoneuron dysfunction between the abductor pollicis brevis (APB) and the abductor digiti minimi (ADM) in amyotrophic lateral sclerosis (ALS) patients based on studying F-waves. Forty ALS patients and 20 normal controls (NCs) underwent motor nerve conduction studies on both median and ulnar nerves, including F-waves elicited by 100 electrical stimuli. The F-wave persistence (*P* < 0.05), index repeating neuron (RN; *P* < 0.001), and index repeater F-waves (Freps; *P* < 0.001) significantly differed between the APB and the ADM in the NC participants. For the hands of the ALS patients that lacked detectable wasting or weakness and exhibited either no or mild impairment of discrete finger movements, significantly reduced F-wave persistence (*P* < 0.001), increased index RN (*P* < 0.001), and increased index Freps (*P* < 0.001) were observed in APB in comparison with the normal participants, with relatively normal ADM F-wave parameters. For the hands of ALS patients that exhibited wasting and weakness, the mean F-wave amplitude (*P* < 0.05), the F/M amplitude ratio (*P* < 0.05), F-wave persistence (*P* < 0.001), index RN (*P* < 0.05), and index Freps (*P* < 0.05) significantly differed between APB and ADM. The differences in the dysfunction of motoneurons innervating APB and ADM are unique manifestations in ALS patients. The F-wave persistence (*P* = 0.002), index RN (*P* < 0.001), and index Freps (*P* < 0.001) in the APB seemed to differentiate ALS from the NCs more robustly than the ADM/APB Compound muscle action potential (CMAP) amplitude ratio. Thus, F-waves may reveal subclinical alterations in anterior horn cells, and may potentially help to distinguish ALS from mimic disorders.

## Introduction

Amyotrophic lateral sclerosis (ALS) is a progressive disorder characterized by the involvement of both upper and lower motor neurons (UMNs and LMNs). In ALS, muscle wasting preferentially affects the abductor pollicis brevis (APB) and first dorsal interosseous, with relative preservation of the abductor digiti minimi (ADM). This specific feature of ALS has been termed the “split-hand” sign (Wilbourn, 2000). In general, the extent of motor unit loss is significantly greater in the APB than in the ADM in patients with ALS (Kuwabara et al., 1999). Both cortical and peripheral mechanisms have been proposed to underlie the different levels of atrophy among the small hand muscles in ALS (Weber et al., 2000; Shibuya et al., 2013). The findings of transcranial magnetic stimulation studies have shown that corticomotoneuronal input to the spinal motoneurons innervating the thenar complex is more extensive in normal participants (Macdonell et al., 1999; Menon et al., 2014). Such a difference in inputs might cause these spinal motoneurons to preferentially degenerate in ALS through a transsynaptic anterograde excitotoxic mechanism. Corticomotoneuronal projections to the thenar complex are preferentially affected in ALS, which suggests that corticomotoneuronal dysfunction contributes to the split-hand sign in ALS (Weber et al., 2000). Studies of peripheral nerve excitability have suggested that the motoneuron axons innervating the APB are hyperexcitable and prone to degeneration in ALS (Vucic and Kiernan, 2010; Shibuya et al., 2013). However, to date, there are few studies that have directly compared the dysfunction or excitability of motoneurons innervating the APB and ADM. The F-wave is a late and low-amplitude response that reflects antidromic activation of motoneurons (Pastore-Olmedo et al., 2009). F-waves may provide an independent measure of segmental motoneuron excitability (Fisher, 1992; Milanov, 1992; Hachisuka et al., 2015). The primary purpose of this study was to examine the differences in dysfunction between spinal motoneurons innervating the APB and ADM in ALS using F-waves.

## Materials and Methods

### Participants

We studied 40 patients with sporadic ALS and 20 age- and gender-matched normal participants. All patients were seen at the Department of Neurology in Peking Union Medical College Hospital between August 2013 and June 2014. The ALS patients who met the modified El Escorial criteria for definite, probable, or probable laboratory-supported ALS (Brooks et al., 2000) were consecutively enrolled in the study. Various electrophysiological examinations and clinical features, such as age at enrollment and the time from symptom onset, were analyzed in these patients. No genetic mutation had been identified in any of the ALS patients. UMN involvement in the upper limbs was suggested by the presence of clonus, increased tone, brisk tendon reflexes, and positive Hoffman signs. The ALS patients were assigned two groups based on their physical symptoms. The patients in group 1 (20 patients with ALS) had wasting and weakness of the intrinsic hand muscles. The patients in group 2 (20 patients with ALS) exhibited no wasting or weakness of the intrinsic hand muscles and showed either no or mild impairment of discrete finger movements. The data for the more affected hand were analyzed for the ALS patients in group 1, whereas, the data for the more intact hand were analyzed for the ALS patients in group 2. The data for the left hand were analyzed for the normal controls (NCs). The ALS patients were clinically staged using the ALS functional rating scale-revised (ALSFRS-R; Cedarbaum et al., 1999) and categorized according to the site of disease onset. The electrodiagnostic features of all ALS patients were consistent with a diffuse and progressive degeneration of anterior horn cells. At the time of the investigation, none of the patients were taking riluzole or other antispasticity drugs. Participants with coincidental carpal or cubital tunnel syndrome based on clinical examination and nerve conduction studies were excluded. All subjects provided informed written consent to participate in the investigation. The study was approved by the Ethics Committee of Clinical Research of Peking Union Medical College Hospital (Beijing, China), and all procedures were conducted in accordance with the Declaration of Helsinki.

### Nerve Conduction Study

The electrophysiological tests were performed using a Viking IV electromyography (EEG) system (Nicolet Biomedical, Madison, WI, USA). Compound muscle action potentials (CMAPs) were recorded from the APB and ADM muscles after median or ulnar nerve stimulation at the wrist. The skin temperature for the studied limbs was maintained at >32°C. The distal motor latency (DML), peak-to-peak CMAP amplitude, motor conduction velocity (MCV) and ADM/APB CMAP amplitude ratio were measured.

### F-wave Study

All subjects were supine and relaxed throughout the experiments. The F-waves of the median and ulnar nerves were recorded with surface electrodes attached to the skin over the APB and ADM muscles. The studied nerves were stimulated by delivering 100 supramaximal stimuli with a frequency of 1 Hz at a site that was 7 cm proximal to the active recording electrode with the cathode proximal to the anode. A total of 100 stimuli were considered appropriate for exploring the full potential of F-waves (Fisher et al., 1994). The filter settings were 20 Hz to 10 kHz, the sweep speed was 5 ms per division, and the amplifier gain was 0.5 mV per division. A-waves, which were defined as identical late responses with constant latencies that occurred in at least 8 out of 20 traces, were excluded from the F-wave study (Puksa et al., 2003). The following F-wave parameters were analyzed: minimal latency, mean latency, maximal latency, F-wave persistence, mean amplitude, mean F/M amplitude ratio, and number of repeater F-waves. The peak-to-peak amplitude of an F-wave was measured if the amplitude was at least 40 μV. The mean F/M amplitude ratio was calculated by dividing the mean F-wave amplitude by the corresponding maximal CMAP amplitude. A repeating neuron (RN) was a neuron that gave rise to a series of F-waves with identical latencies, amplitudes, and shapes, and these F-waves were defined as repeater F-waves. RNs and repeater F-waves were detected via visual inspection and were manually superimposed on the other repeater F-waves. The repeater F-waves were measured using the following indices: index RN = 100 × number of RN/number of traces with different F-wave shapes in a series of 100 stimuli; index repeater F-waves (Freps) = 100 × number of repeater F-waves/total number of traces with F-waves in the same nerve (Chroni et al., 2012).

### Statistical Analyses

The Shapiro–Wilk test was used to assess the normality of the data. When the *P*-value in the analysis of variance achieved significance, the Student–Newman–Keuls test was performed. The independent-sample *t*-test was used to assess differences between two groups. For non-parametric data, comparisons between groups were performed using the Kruskal–Wallis *H* test. Once the null hypothesis was rejected, pairwise comparisons of the groups were tested using the Mann–Whitney *U* test and Bonferroni correction with a significance level of *P* < 0.017. The differences in categorical variables were examined by the Chi-square test. The statistical significance was set at *P* < 0.05. SPSS for Windows, version 21.0 (SPSS, Inc., Chicago, IL, USA), was used to perform the statistical analyses.

## Results

The clinical profiles of the ALS patients and the NC participants are summarized in Table 1. All the ALS patients were clinically LMN-predominant. The age at examination, gender ratio and height were comparable across the three groups. Among the ALS patients, upper limb-onset disease accounted for 70% of the patients in group 1 and 25% of the patients in group 2. There were 11 ALS patients in group 1 and 10 in group 2 with two affected body regions, and there were 9 generalized patients in group 1 and 10 in group 2. The disease duration and the ALSFRS-R scores did not differ significantly between the ALS patients in groups 1 and 2.

**Table 1:** **Clinical profiles of the participants**.

Parameters	Group 1	Group 2	NC	*P*-value Group1 vs. NC	*P*-value Group 2 vs. NC	*P*-value Groups 1 vs. 2
Age	47.15 ± 9.56	49.6 ± 8.93	50.9 ± 10.86	0.461	0.862	0.445
[years ± SD (range)]	(28–68)	(33–65)	(35–65)
Gender (male: female)	16:4	12:8	12:8	0.168	1	0.168
Disease duration	14.30 ± 6.59	15.10 ± 15.77	NA	NA	NA	0.343
[months ± SD (range)]	(6–26)	(3–72)
Height (cm)	169.35 ± 6.49	168.9 ± 6.54	167.65 ± 7.71	>0.05	>0.05	>0.05
ALSFRS-R score	38.20 ± 6.65	40.30 ± 3.33	NA	NA	NA	0.478
[mean ± SD (range)]	(17–44)	(34–45)

*ALSFRS-R, amyotrophic lateral sclerosis functional rating scale-revised; NA, not applicable; NC, normal control; SD, standard deviation. All data are expressed as mean ± SD*.

**Figure 1:**
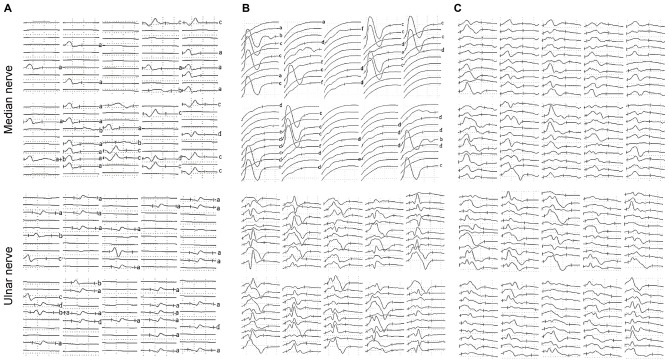
**Representative examples of F-waves recorded from the amyotrophic lateral sclerosis (ALS) patients and the normal controls (NCs). (A)** F-waves recorded from an ALS patient hand exhibiting wasting and weakness. Reduced F-wave persistence and increased number of repeater F-waves were observed on F-waves recorded from both the median and ulnar nerves. **(B)** F-waves recorded from an ALS patient hand lacking detectable wasting or weakness. The F-wave persistence was reduced and the number of repeater F-waves was increased on the median nerve, while parameters of F-waves recorded from the ulnar nerve were relatively normal. **(C)** F-waves recorded from a healthy subject on the left upper limb. F-wave amplitudes, latencies and waveforms were variable, and F-wave persistence was normal on both the median and ulnar nerves. Letters to the right of record identify repeater F-waves on the basis of amplitude, latency and waveform. Calibrations are 0.5 mV and 5 ms for F-wave recording.

Table 2 shows the results of the nerve conduction study for the ALS patients and the NCs. The ADM/APB CMAP amplitude ratio was significantly increased in the ALS patients in group 1 compared with the NC group, which was observed consistently with the split-hand sign in ALS patients (Kuwabara et al., 2008). The results of the F-wave study for the ALS patients and NCs are shown in Table 3. The mean F-wave amplitude for the APB was significantly lower in group 1 than in group 2, which were comparable between group 2 and the NCs. The mean F-wave amplitudes of the ADM in groups 1 and 2 were significantly increased compared to that in the NCs. The F/M amplitude ratios of the APB and ADM in group 1 were significantly higher than those in group 2 and NCs, whereas group 2 and NCs showed similar F/M amplitude ratios. The F-wave persistence of the APB, which was significantly lower in the ALS patients than in NCs, was comparable between groups 1 and 2. The F-wave persistence of the ADM was significantly lower in group 1 than in group 2 and NCs, whereas the F-wave persistence of the ADM was similar between group 2 and NCs. The index RN and index Freps of APB were significantly increased in groups 1 and 2 compared with the NCs, but these indices were comparable between groups 1 and 2. The index RN and index Freps of the ADM was significantly higher in group 1 than in group 2 and the NCs, whereas group 2 and NCs showed similar values for these indices. Figure 1 presents representative examples of F-wave traces for the median and the ulnar nerves recorded from ALS patients in groups 1, 2 and NCs.

**Table 2:** **Results of nerve conduction study**.

Parameters	Group 1	Group 2	NC	*P*-value Group 1 vs. NC	*P*-value Group 2 vs. NC	*P*-value Groups 1 vs. 2
DML (ms)
APB	3.71 (0.78)*	3.17 (0.40)*	2.93 (0.41)*	**<0.001**	0.028	**<0.001**
ADM	2.46 (0.46)*	2.29 (0.31)*	2.15 (0.29)*	**0.002**	0.142	0.301
CMAP amplitude (mV)
APB	2.85 (1.39)*	11.76 (2.84)	12.44 (3.21)	**<0.05**	>0.05	**<0.05**
ADM	8.34 (2.58)*	13.25 (2.46)	13.76 (2.31)	**<0.05**	>0.05	**<0.05**
ADM/APB ratio	3.74 (2.56)	1.12 (0.28)	1.12 (0.39)	**<0.001**	0.620	**<0.001**
MCV (m/s)
APB	55.56 (5.78)	56.99 (5.58)	57.13 (5.98)	>0.05	>0.05	>0.05
ADM	56.53 (6.87)	58.35 (6.78)	56.33 (3.51)	>0.05	>0.05	>0.05

ADM, abductor digiti minimi; ALS, amyotrophic lateral sclerosis; APB, abductor pollicis brevis; ADM/APB ratio, ADM/APB CMAP amplitude ratio; CMAP, compound muscle action potential; DML, distal motor latency; MCV, motor conduction velocity; NC, normal control. All data are expressed as mean (SD). Values with significant differences printed in bold characters. *P < 0.001, between the APB and the ADM in each group. For comparisons of DML and ADM/APB ratio between the ALS patients in groups 1 and 2, and the normal controls, Bonferroni correction with a level of significance of <0.017 was used.

**Table 3:** **F-wave study in the ALS patients and the normal controls**.

	Group 1	Group 2	NC	*P*-value Group 1 vs. NC	*P*-value Group 2 vs. NC	*P*-value Groups 1 vs. 2
Minimal F latency (ms)
APB	25.61 (3.17)	24.55 (1.86)	23.89 (1.53)	>0.05	>0.05	>0.05
ADM	25.85 (2.21)	24.54 (1.55)	24.06 (1.89)	**<0.05**	>0.05	**<0.05**
Maximal F latency (ms)
APB	29.98 (2.64)	28.74 (1.87)	26.94 (1.81)	**<0.05**	**<0.05**	>0.05
ADM	30.18 (2.31)	27.99 (2.02)	26.77 (2.04)	**<0.05**	>0.05	**<0.05**
Mean F latency (ms)
APB	27.77 (2.43)	26.14 (1.67)	25.23 (1.65)	**<0.05**	>0.05	**<0.05**
ADM	27.45 (2.27)	25.99 (1.67)	25.26 (1.97)	**<0.05**	>0.05	**<0.05**
Mean F amplitude (μV)
APB	263.05 (287.06)**	342.75 (164.99)	262.70 (91.83)	>0.05	>0.05	**<0.05**
ADM	375.90 (146.46)**	350.20 (118.38)	267.40 (78.83)	**<0.05**	**<0.05**	>0.05
F/M amplitude ratio
APB	13.35 (19.76)**	3.15 (1.95)	2.15 (0.74)	**<0.001**	0.063	**<0.001**
ADM	4.85 (2.54)**	2.71 (1.07)	2.01 (0.74)	**<0.001**	0.020	**<0.001**
F-wave persistence (%)
APB	39.35 (27.76)*	51.00 (17.45)*	95.40 (4.90)**	**<0.001**	**<0.001**	0.076
ADM	78.70 (23.32)*	98.10 (3.39)*	99.25 (1.45)**	**<0.001**	0.327	**<0.001**
Index RN (%)
APB	41.89 (26.09)**	31.87 (17.47)*	3.44 (2.98)*	**<0.001**	**<0.001**	0.277
ADM	25.26 (16.59)**	2.19 (5.44)*	0.70 (1.59)*	**<0.001**	0.157	**<0.001**
Index Freps (%)
APB	72.50 (23.43)**	62.65 (18.86)*	7.07 (6.16)*	**<0.001**	**<0.001**	0.134
ADM	51.27 (30.09)**	4.67 (11.19)*	1.39 (3.19)*	**<0.001**	0.149	**<0.001**

APB, abductor pollicis brevis; ADM, abductor digiti minimi; NC, normal control. All data are expressed as mean (SD). Values with significant differences printed in bold characters. *P < 0.001, **P < 0.05, between the APB and the ADM in each group. For comparisons of F/M amplitude ratio, F-wave persistence, index RN, and index Freps between the ALS patients in groups 1 and 2, and the normal controls, Bonferroni correction with a level of significance of <0.017 was used.

Table 4 shows the diagnostic performance of the F-wave in ALS vs. NCs. The F-wave could be used to help differentiate ALS patients from NCs. The index RN and index Freps of the APB appeared to be reliable variables for differentiating ALS patients from NCs, as the area under the curve (AUC) for the index RN (0.998, 95% confidence interval (CI) 0.937–1.000) and index Freps (1.000, 95% CI 0.940–1.000) showed “very good” diagnostic utility. The remainder of the F-wave variables had lower AUC values than the index RN and index Freps of the APB and thus showed less diagnostic utility. The ADM/APB CMAP amplitude ratio showed an AUC of 0.766 (95% CI 0.638–0.865, *P* < 0.001) and could moderately differentiate ALS patients from NCs. Using a cut-off value of ADM/APB > 1.7 (Kim et al., 2015) for diagnosing ALS yielded a moderate sensitivity (52.5%) and a high specificity (85.0%) compared with controls. The F-wave persistence (*P* = 0.002), index RN (*P* < 0.001) and index Freps (*P* < 0.001) in the APB appeared to differentiate ALS patients from NCs more robustly than the ADM/APB CMAP amplitude ratio.

**Table 4:** **Diagnostic performance of F-wave in amyotrophic lateral sclerosis (ALS) vs. normal controls (NCs)**.

Parameters	Cut-off value	Sensitivity (%)	Specificity (%)	AUC (95% CI)
**APB**
Minimal F latency	24.55 ms	60.0	75.0	0.646 (0.505–0.787) *P* = 0.068
Maximal F latency	28.30 ms	70.0	75.0	0.792 (0.678–0.906) *P* < 0.001
Mean F amplitude	260.00 μV	62.5	55.0	0.526 (0.393–0.656) *P* = 0.738
F/M amplitude ratio	2.60	72.5	85.0	0.820 (0.699–0.907) *P* < 0.001
F persistence	81.00%	95.0	100.0	0.976 (0.898–0.998) *P* < 0.001
Index RN	9.64%	97.5	100.0	0.998 (0.937–1.000) *P* < 0.001
Index Freps	19.35%	100.0	100.0	1.000 (0.940–1.000) *P* < 0.001
**ADM**
Minimal F latency	22.80 ms	92.5	35.0	0.664 (0.530–0.781) *P* = 0.034
Maximal F latency	26.70 ms	85.0	65.0	0.769 (0.642–0.868) *P* < 0.001
Mean F latency	26.70 ms	65.0	70.0	0.695 (0.563–0.807) *P* = 0.011
Mean F amplitude	320.00 μV	57.5	85.0	0.716 (0.585–0.825) *P* = 0.001
F/M amplitude ratio	2.66	62.5	90.0	0.819 (0.699–0.907) *P* < 0.001
F persistence	97.00%	55.0	95.0	0.750 (0.621–0.853) *P* < 0.001
Index RN	0.00%	75.0	80.0	0.804 (0.681–0.895) *P* < 0.001
Index Freps	0.00%	75.0	80.0	0.805 (0.682–0.896) *P* < 0.001

*APB, abductor pollicis brevis; ADM, abductor digiti minimi; AUC, area under the curve; CI, confidence interval*.

## Discussion

The primary aim of this study was to use F-wave measures to ascertain the differences in dysfunction between APB and ADM motoneurons in patients with ALS. According to a proposed staging system for ALS (Roche et al., 2012), the stages of the disease were comparable between groups 1 and 2. The difference in the percentage of upper limb onset between groups 1 and 2 may underlie a different pattern of disease progression between the two groups. The estimated number of motor units and CMAP amplitudes are useful parameters for evaluating motoneuron loss, whereas the F-wave may be a direct probe of dysfunction or instability in anterior horn cells (Hachisuka et al., 2015). The F-wave persistence is related to the number of LMNs and motoneuron excitability (Schiller and Stalberg, 1978; de Carvalho et al., 2002; Argyriou et al., 2006). The proposed mechanisms underlying repeater F-waves are increased excitability in particular anterior horn cells, decreased excitability in some motoneurons, or motoneuron loss (Schiller and Stalberg, 1978; Petajan, 1985; Peioglou-Harmoussi et al., 1987; Hachisuka et al., 2015). As motoneurons are lost, repeater F-waves from individual motoneurons may be recognized more easily; however, it has been argued that the low frequency of backfiring of individual motoneurons makes the mechanism rather unlikely (Chroni et al., 2012). Physiologically, there were significant differences in the F-wave persistence and the number of repeater F-waves between the APB and ADM. These findings may be associated with a lower number of functional motoneurons innvervating the APB (Gooch et al., 2014), or increased cortical inhibitory modulation of the APB (Menon et al., 2014).

In ALS, dysfunction of spinal motoneurons develops gradually before the onset of overt symptoms (Bradley, 1987). In the present study, the ALS patients who lacked detectable wasting or weakness in the hands showed significantly reduced F-wave persistence and higher index RN and index Freps values for the APB compared with those of NCs. Furthermore, these ALS patients showed relatively normal F-wave values in the ADM. These data are consistent with a preferential dysfunction of spinal motoneurons innervating the APB in ALS (Baumann et al., 2012). In ALS, however, the spinal motoneurons innervating the APB may be more active than those innervating the ADM, and the CMAP amplitude may not have sufficient sensitivity for detecting motoneuron loss because the remaining motoneurons may provide compensatory collateral reinnervation of denervated muscle fibers (van Dijk et al., 2010). Analyzing F-waves, especially F-waves in the median nerves, could help to detect subtle alterations of anterior horn cells even in ALS patients without clinical symptoms and thus may provide a helpful approach for evaluating the disease progression.

LMN damage has been shown to reduce the amplitude of F-waves (Fisher, 1992). Muscle atrophy may lead to a weaker muscle response by partially neutralizing the hyperexcitability of the motoneuron pool (Drory et al., 1993). In the present study, the ALS patients who presented with the split-hand sign showed significantly reduced F-wave amplitudes, reduced F-wave persistence, and an increased number of repeater F-waves in the APB compared with the ADM, which likely reflects more severe damage to the spinal motoneurons innervating the APB. The formation of large post-reinnervation motor units could contribute to increases in the F-wave amplitude (Drory et al., 2001). A significantly increased F-wave amplitude in the ADM compared with the APB in the ALS patients is consistent with a slower motoneuron loss in the ADM motoneuron pool (Baumann et al., 2012). The F/M amplitude ratio quantifies the proportion of the motoneuron pool that is activated during a series of F-waves (Drory et al., 2001). The significantly increased F/M amplitude ratio in the APB compared with that in the ADM of ALS patients reflects an increased tendency of the motoneurons innervating the APB to generate F-waves. Conversely, the combination of the decreased mean F-wave amplitude and the increased F/M amplitude ratio in the APB emphasizes the preferential involvement of the APB in ALS. Repeater F-waves indicate pathological changes in motor units (Hachisuka et al., 2015). The significant increase in the number of repeater F-waves in the APB may imply a greater degree of hyperexcitability within the spinal motoneurons innervating the APB (Fang et al., 2015).

Intriguingly, ALS patients who lacked detectable hand muscle atrophy showed a mean F-wave amplitude for the APB that was significantly higher than that for the ALS patients who presented with the split-hand sign. However, the mean F-wave amplitude for the ADM was comparable between the two ALS patient groups, regardless of hand muscle atrophy, but was significantly higher in the ALS patients than in the NCs. The differences in the F-wave characteristics between the APB and ADM may be attributed to the competing effects of degeneration and regeneration within the motor unit. With the degeneration of motor neurons, the surviving motor neurons compensate by reinnervating the denervated muscle fibers through axonal sprouting (Ibrahim and el-Abd, 1997). In ALS, the denervation process may begin earlier and progress more rapidly in the thenar region. The rate of degeneration was slower in the motoneurons innervating the ADM than in those innervating the APB. Further studies are necessary to elucidate the mechanisms in ALS that contribute to the differences in motoneuron dysfunction between motoneurons innervating the APB and ADM. We suspected that cortical mechanisms, peripheral axonal mechanisms, or spinal segmental dysfunction, particularly in spinal inhibitory circuits, might contribute to the preferential degeneration of spinal motoneurons innervating the APB (Turner and Kiernan, 2012; Ramírez-Jarquín et al., 2014).

Previous research demonstrated that the split-hand sign is more frequently observed in ALS, and the increased ADM/APB CMAP amplitude ratio is nearly specific to ALS. The present study demonstrated that the neurophysiological criterion for the ADM/APB of a CMAP amplitude ratio >1.7 (Kuwabara et al., 2008) has a moderate sensitivity and high specificity in differentiating ALS from NCs, which is consistent with previous studies (Kim et al., 2015). In this study, F-wave parameters could help to reliably differentiate ALS patients from the normal participants. Furthermore, the F-wave persistence, index RN and index Freps in the APB could reliably differentiate ALS patients from the NCs, as these measures showed larger AUC values than did the ADM/APB CMAP amplitude ratio.

Our analysis has several limitations. This was a cross-sectional study, and a relatively small number of participants were included. A follow-up study with a larger population is necessary to specifically determine the extent of spinal dysfunction associated with the split-hand sign in ALS. Another potential limitation is the lack of a patient control group consisting of patients with syndromes that mimic ALS. Such a group would be useful to assess the clinical value of the F-wave in discriminating ALS from disorders that mimic ALS. Moreover, further electrophysiological studies that use the methods for evaluating UMN dysfunction, as well as the excitability of the motor axons, should be performed on the same patient groups to shed more light on the pathophysiology of this phenomenon.

In conclusion, our findings demonstrate differences in F-wave characteristics between the APB and ADM in patients with ALS. These alterations in the F-waves are characteristic of ALS patients and may be helpful for differentiating ALS from certain disorders that mimic ALS. Elucidating the pathophysiological mechanisms underlying the differing levels of atrophy in the small hand muscles would shed light on the pathogenesis of ALS.

## Author Contributions

JF and LC: conceived, performed and designed the experiments. JF, LC, ML and YG: analyzed the data. ML, YG, XL, DL, BC, DS and QD: contributed reagents/materials/analysis tools. JF and LC: contributed to the writing of the manuscript.

## Conflict of Interest Statement

The authors declare that the research was conducted in the absence of any commercial or financial relationships that could be construed as a potential conflict of interest.
